# Synergistic effects of elastic band and vibration training on muscle strength, balance, and mobility in older women with a history of falls: a randomised controlled trial

**DOI:** 10.3389/fbioe.2025.1525000

**Published:** 2025-04-03

**Authors:** Zhou Zhang, Weizhi Xiong, Heng Liu

**Affiliations:** ^1^ BAYI Orthopedic Hospital, China RongTong Medical Healthcare Group Co. Ltd., Chengdu, China; ^2^ School of Physical Education, Chengdu Sport University, Chengdu, China; ^3^ College of Physical Education, Chongqing University, Chongqing, China

**Keywords:** exercise intervention, muscle strength enhancement, balance improvement, physical performance, fall prevention

## Abstract

**Objective:**

This study aimed to investigate the effects of combined elastic band resistance training (EBRT) and whole-body vibration training (WBVT) on lower limb isokinetic muscle strength, balance and physical performance in older women with history of falls.

**Methods:**

A total of 102 older women with a history of falls was randomly assigned to the elastic band group (*n* = 28), vibration group (*n* = 28), elastic band plus vibration group (*n* = 28) and control group (*n* = 28) for a 12-week program. All groups performed identical exercises (half squats, static squats, left/right lunges) thrice weekly. The control group trained on a flat ground without elastic bands or vibration; the elastic band group used elastic bands on the flat ground, while the vibration and combined groups exercised on a vibration platform. Training parameters included 3 sets × 10 repetitions with 20-s rest intervals.

**Results:**

Post-intervention, knee flexion peak torque increased by 9.7% (*p* < 0.05). Total length (TL), elliptical area (EA), total offset index (TAI), timed up and go test (TUG) and five times sit-to-stand test (FTSST) decreased by 16.2%, 14.2%, 21.9%, 16.9% and 14.0%, respectively; and modified fall efficacy scale (MFES) increased by 17.6% (*p* < 0.01) in the elastic band group. Knee extension peak torque increased by 16.3% (*p* < 0.05). TL, EA, TAI, TUG and FTSST decreased by 14.8%, 12.2%, 18.9%, 12.3% and 11.5%, respectively; and MFES increased by 16.2% in the vibration group (*p* < 0.01). Hip and knee flexion and knee and ankle extension peak torque increased by 19.5%, 18.8%, 30.2% and 30.1% (*p* < 0.01), respectively, in the elastic band plus vibration group. TAI, TL, EA, TUG and FTSST decreased by 28.5%, 24.6%, 38.3%, 22.4% and 29.0%, respectively, and MFES increased by 42.1% in the elastic band plus vibration group (*p* < 0.01). The elastic band plus vibration group demonstrated greater improvements in knee and ankle strength compared to single interventions, while the elastic band and vibration groups showed smaller changes (*p* < 0.05). Additionally TL, EA, TAI, TUG, and FTSST all decreased, while MFES increased (*p* < 0.05).

**Conclusion:**

The combined EBRT and WBVT can enhance knee and ankle isokinetic muscle strength, improve balance ability and physical performance and reduce fear of falls by a greater degree than single interventions. EBRT and WBVT had limited effects on improving lower limb isokinetic muscle strength but enhanced balance ability, physical performance, and reduced fear of falls in older women with a history of falls. Both training modalities showed similar effects.

## Introduction

Falling and fear of falling do not only depend on balance but are multifactorial (Tabacchi et al., 2025). Balance ability is an important fundamental function for maintaining daily activities, such as standing, walking, and climbing stairs (Chang et al., 2024a), and it is correlated with age. Individuals aged 21–50 years typically exhibit stable balance, which gradually declines with age. The decline is particularly pronounced in individuals over the age of 70 years and leads to an increased risk of falling (Sturnieks et al., 2008). A follow-up study on 5,131 men and women aged 40–95 years indicated that women are more prone to falls (Nordström et al., 2011). In a report by the Center for Disease Control and Prevention in the United States, it is indicated that approximately 33% of older adults (aged 65–75) experience at least one fall per year, which is defined as having a history of falls. Furthermore, the probability of those older adults with a history of falls experiencing another fall within the subsequent year is approximately 50% (Rubenstein, 2006). A survey of 16,393 Chinese adults aged 65 years and above revealed that approximately 14% had a history of falls within 12 months, and the proportion of women was 25% higher than that of men (Zhang et al., 2019). Falls can result in soft tissue injuries, joint dislocation, fractures or even head injuries, leading to substantial medical expenses for families (Vaishya and Abhishek, 2020; Afridi et al., 2021). Therefore, improving balance ability and preventing recurrent falls in older adults with a history of falls are important issues worthy of attention.

Regular exercise can increase balance ability in older adults (Kim et al., 2024; Andriana and Nurdianto, 2022), and resistance exercise is an effective measure for fall prevention (Sherrington et al., 2020; Fragala et al., 2019). Common exercise methods include dumbbell exercise (Sherrington et al., 2020; Kassiano et al., 2022) or elastic band resistance training (EBRT) (Banitalebi et al., 2020; Gargallo et al., 2024). Elastic bands are convenient to carry, can be easily adjusted according to exercise load and offer a variety of movements and are thus suitable for older adults (Banitalebi et al., 2020). EBRT has positive effects on muscle strength (Gargallo et al., 2024), balance ability (Kwak et al., 2016), proprioception (Areas et al., 2013) and physical performance (Oesen et al., 2015) in older adults. Whole-body vibration training (WBVT) (Van Heuvelen et al., 2021) is suitable for older adults or individuals who have low physical activity (Cheng et al., 2021a). It involves standing on a vibrating platform and using mechanical vibration and load to stimulate the body (Cheng et al., 2022a). WBVT effectively improves posture control by increasing joint muscle strength (Cheng et al., 2022b; Gonçalves et al., 2023), balance ability (Liu et al., 2023) and proprioception (Trans et al., 2009) and reducing neuro-muscular reaction time (Monteiro et al., 2022), thereby reducing the risk of falls. However, current research into the individual effects of EBRT or WBVT on older adults remain limited, and literature exploring whether combined training with EBRT and WBVT has a synergistic effect that improve posture control in older adults and whether the effects of these training methods differ is lacking. Therefore, the present study aims to examine the effects of 12 weeks of EBRT, WBVT, and combined training, against a non-training control group, on lower limb isokinetic muscle strength, balance ability and physical performance in older women with a history of falls. We hypothesize that the combined training program would lead to the largest beneficial changes.

## Materials and methods

### Participants

This study was a randomized controlled trial (four-arm parallel design, assessor-blinded). The data statisticians were blinded to the group allocation. This study obtained approval from the human experiment ethics committee of Chengdu Sport University (No: 202206). Participants were recruited in Chengdu, Wuhou District, through questionnaires and interviews, and older women with a history of falls within the past 2 years were targeted. The inclusion criteria were as follows: females aged 60–70 years; at least one accidental fall in the past year; passing a cardiac function examination; willingness to engage in fitness activities; and signing an informed consent form in accordance with the Helsinki Declaration. The exclusion criteria were as follows: engaging in regular exercise using other methods within the past 6 months; a history of severe lower limb trauma; epilepsy or movement disorders; and having a pacemaker. Withdrawal criteria included: voluntary withdrawal due to personal reasons; emergence of severe medical conditions (e.g., cardiovascular events, fractures); consecutive absence from training sessions ≥3 times. Contraindications for participation/training termination: occurrence of dizziness, chest pain, or acute joint injury during training.

**FIGURE 1:**
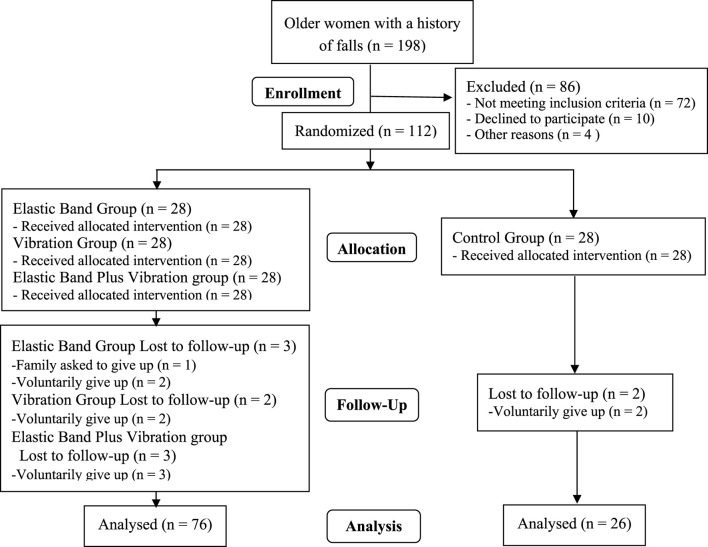
Participant selection flow diagram.

Using previous data on the effects of EBRT (Gargallo et al., 2024; Kwak et al., 2016) and WBVT (Liu et al., 2023) on muscle strength and balance ability in older women, we calculated the sample size needed by using G-Power software. At least 110 participants were needed because of the four-group two-measurement experimental design. The effect size was 0.3, power was 0.8 and α = 0.05 and estimated sample loss was approximately 10%. Initially, 112 participants were recruited using random digit allocation (28 ones, 28 twos, 28 threes, 28 fours) and were distributed into the elastic band group (n = 28), vibration group (n = 28), elastic band plus vibration group (n = 28) and control group (n = 28). During the study period, 10 participants (elastic band group: three cases; vibration group: two cases; elastic band plus vibration group: three cases; control group: two cases) dropped out for personal reasons. The sample loss rate was 8.9%, and 102 participants completed the entire process (Table 1; Figure 1). No differences were found among groups with regard to age, height and body mass (p > 0.05).

**TABLE 1:** Demographic characteristics of the participants at baseline.

Group	*n*	Age (years)	Height (cm)	Body mass (kg)	Periods (weeks)	Number of falls in the last 1 year (times)		
						1	2	≥3
Elastic band group	25	64.5 (3.4)	158.5 (6.7)	61.7 (5.5)	12	14	8	3
Vibration group	26	64.7 (4.2)	157.9 (5.8)	61.8 (4.8)	12	15	9	2
Elastic band plus vibration group	25	65.2 (3.0)	158.4 (5.0)	62.4 (5.0)	12	13	9	3
Control group	26	65.0 (3.9)	158.3 (4.9)	61.9 (6.2)	12	15	9	2

### Elastic band resistance training and whole-body vibration training

The four groups completed the same number of sets and repetitions for four exercises. The exercise protocol was performed for 12 weeks, with three sessions per week. The four exercises included half squats, static weight-free squats, and left and right lunge squats. Each exercise was performed for three sets of 10 repetitions, with a 20-s rest between sets. Specifically, half squats and static weight-free squats were held for 20 s per set, with a 10-s interval between sets; left and right lunge squats were performed 10 times per set, with a 20-s rest between sets (Xiong and Liu, 2023). The overall training protocol followed a 3 × 10 repetition scheme with approximately 20 s of rest between sets (Silva et al., 2023). The control group performed the same four exercises on a flat ground without elastic bands or vibration. The elastic band group used elastic bands during the exercises on a flat ground, while the elastic band plus vibration group performed the exercises on a vibrating platform (with the device turned on). The vibration group exercised on the vibrating platform without elastic bands.

For EBRT, the participants in the elastic band group and elastic band plus vibration group used green elastic bands (Joinfit, 2,000 mm × 150 mm × 0.45 mm; Joinfit Inc., Irvine, CA, United States) for the four exercises. Elastic band resistance training (EBRT) incorporated both concentric contractions (muscle shortening against resistance) and eccentric contractions (muscle lengthening against resistance). When stretching the elastic band up to about two times the original length, the four exercises were completed. For WBVT, participants in the vibration group and elastic band plus vibration group used a WBVT device (Power-Plate, dimensions: 96 cm × 96 cm × 156 cm; Power-Plate International, Irvine, CA, United States). The parameters used for WBVT were based on the studies of Cheng et al. (2022a), including a frequency of 45 Hz and amplitude of 3 mm. The participants performed all four exercises (the training protocol is consistent as described above) on the vibration platform during the WBVT sessions, which lasted approximately 15–20 min per session.

### Dependent variables

The primary outcomes included lower limb isokinetic muscle strength, balance ability, physical performance, and fear of falling. Assessments were conducted as follows.

### Isokinetic muscle strength testing

Isokinetic muscle strength testing was conducted using an IsoMed 2000 isokinetic dynamometer from Germany. Bilateral hip, knee and ankle joint flexion or extension strength tests were performed at a speed of 60°/s for three repetitions at baseline and after 12 weeks. The participants were in a supine position for hip and ankle joint testing and in a sitting position for knee joint testing. The ranges of motion for the hip, knee and ankle joints were set at 120°, 80° and 70°, respectively. Peak torque was selected as the isokinetic muscle strength testing parameter and represented the maximum output torque (Nm) produced by muscle contraction during the entire joint movement. It reflects the participants’ strength capacity (Gonçalves et al., 2023; Cheng et al., 2021b).

### Balance ability testing

The Pro-Kin254 Balance Testing System from Italy (Tecnobody) was used. Prior to formal testing, the participants underwent a pre-test to familiarise themselves with the testing procedure. Static balance was assessed by having participants stand barefoot on the testing platform while maintaining their body still for 30 s. The system recorded changes in the centre of pressure (COP), from which total length (TL) and elliptical area (EA) of COP oscillations were calculated. High values indicate poor static balance ability (Zhang et al., 2024). Dynamic balance was assessed by having participants stand on both legs on the platform (the test platform is not fixed and will randomly tilt 5° to the left, right, front and back directions) and attempt to keep their trunk as still as possible for 30 s. If the participants shifted their feet on the platform or held onto the railing during the test, then it was considered a test failure, and they were required to repeat the test. The dynamic posture stability index used was the total offset index (TAI), which represents the angle formed between whole-body displacement and a vertical midline. High values indicate poor dynamic posture stability (Zhang et al., 2024).

### Physical performance testing

The timed up and go test (TUG) and five times sit-to-stand test (FTSST) were adapted from the Senior Fitness Test to assess functional mobility (Rikli and Jones, 2013). The TUG required participants to sit in a chair with their feet shoulder-width apart and then stand up as quickly as possible, walk to a mark 3 m away from the chair, turn around, return to the chair and sit down again. The time taken to complete the task was recorded, and three trials were performed with a 30-s interval between each trial. A short completion time indicated good ability in walking tasks (Beauchet et al., 2011). The FTSST required participants to sit on an armless and backless chair and then repeatedly stand up and sit down as quickly as possible for five times. The time taken from the start of the first head movement to the maximum hip extension of the fifth repetition was recorded. Three trials were performed with a 30-s interval between each trial, and the average time was calculated. A short completion time indicates good ability in postural control (Muñoz et al., 2021).

### Fear of falling

The modified fall efficacy scale (MFES) consists of 14 items rated on an 11-point scale from 0 to 10. This scale reflects the fall efficacy and fall fear, and a low score indicates low confidence and increased fear of falling. Its internal consistency (Cronbach’s α = 0.94) and test–retest reliability (ICC = 0.89–0.95) are excellent (Kwan et al., 2013).

### Statistical analysis

Statistical analysis was performed using IBM SPSS Statistics software (Version 19.0; IBM Corp., Armonk, NY, United States). The Shapiro–Wilk test was conducted separately for each group to assess data normality. Effect sizes were calculated using Cohen’s d for within-group comparisons, with values of 0.2, 0.5, and 0.8 indicating small, medium, and large effects, respectively. Two-way analysis of variance (ANOVA) was performed for group (4) × time (2) main effects analysis and analysis of the interaction effects of group and time factors. When an interaction effect was found, follow-up analyses were conducted to analyse the main effect of time or group; when no interaction effect was found, follow-up analyses were conducted to analyse either the main effect of group or time (Cheng et al., 2024). Post-hoc comparisons among different groups were made using Bonferroni correction, and the significance level was set at 0.05 (α).

## Results

### Participant flow and baseline characteristics

**FIGURE 2:**
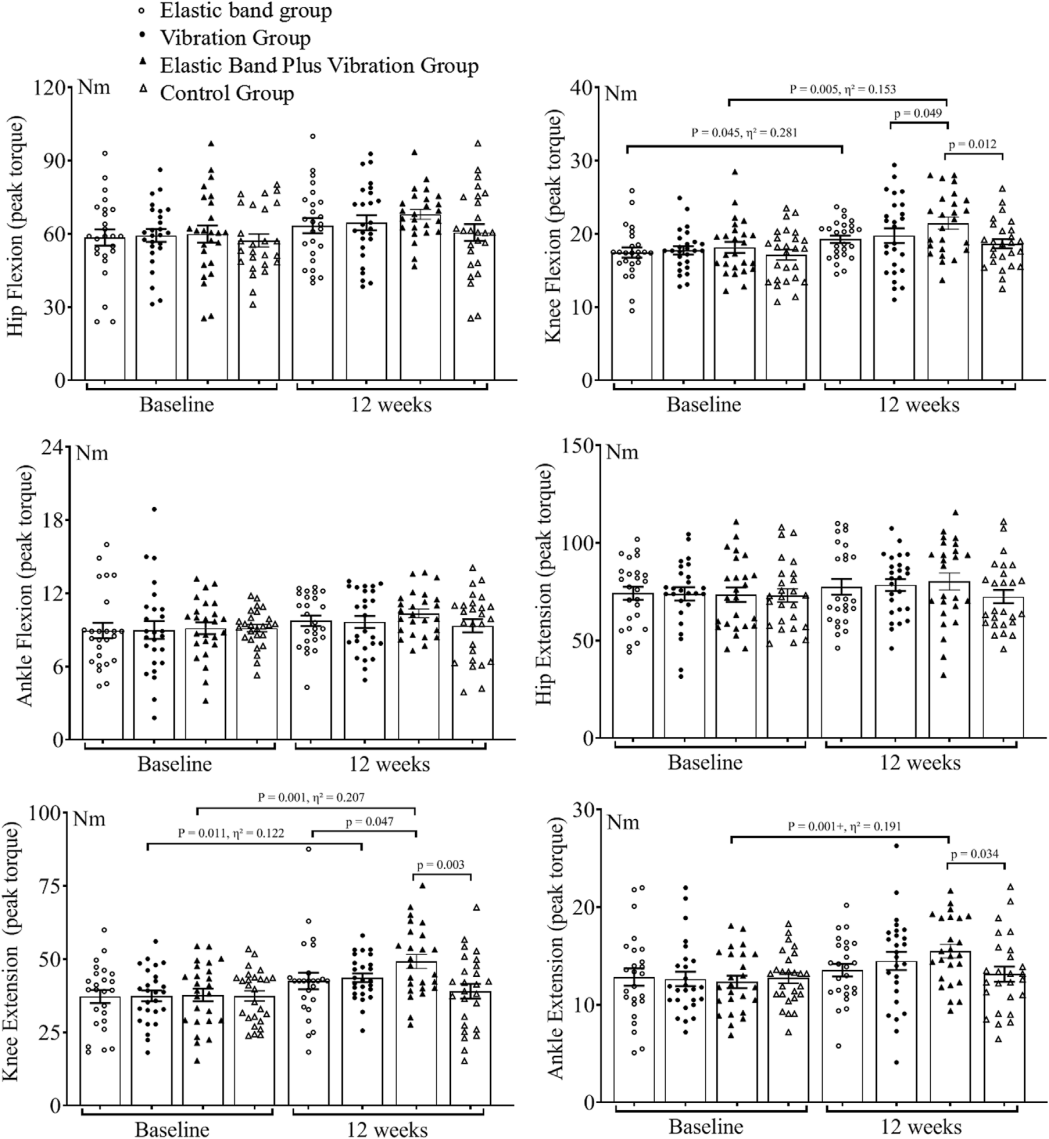
Isokinetic muscle strength test of hip, knee and ankle test results.

**FIGURE 3:**
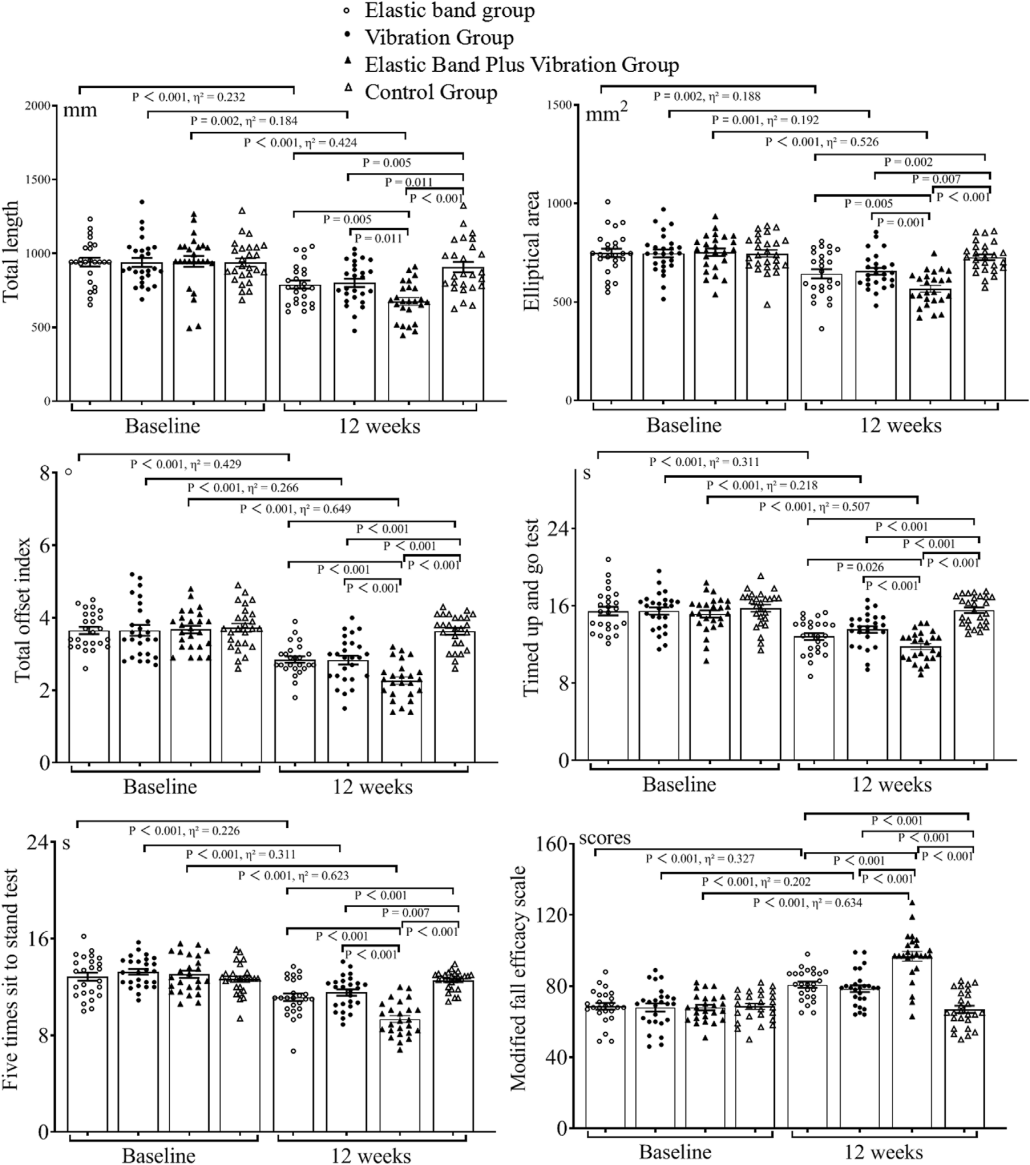
Balance performance, exercise performance, and Modified fall efficacy scale test results.

Among the 112 participants who were initially randomized, 102 finished the study, resulting in an attrition rate of 8.9% (Figure 1). There were no significant differences in baseline characteristics between those who completed the study and those who dropped out (*p* > 0.05). The demographic data can be found in Table 1. The baseline and 12-week test results for the four groups are presented in Tables 2 and 3 and illustrated in Figures 2, 3.

**TABLE 2:** Isokinetic muscle strength test of hip, knee and ankle test results.

Group	Weeks	Flexion (Nm)			Extension (Nm)		
		Hip	Knee	Ankle	Hip	Knee	Ankle
Elastic band group	0	58.5 ± 16.6	17.5 ± 3.5	8.9 ± 3.1	74.3 ± 16.7	37.3 ± 11.2	12.9 ± 4.5
	12	63.4 ± 15.0	19.2 ± 2.6	9.8 ± 2.2	77.6 ± 20.2	42.6 ± 13.9	13.6 ± 3.3
Vibration group	0	59.3 ± 13.2	17.7 ± 2.8	9.0 ± 3.7	73.9 ± 17.0	37.5 ± 9.4	12.7 ± 3.8
	12	64.5 ± 15.6	19.7 ± 5.2	9.7 ± 2.6	78.0 ± 15.0	43.6 ± 8.0	14.0 ± 4.8
Elastic band plus vibration group	0	57.0 ± 13.2	18.1 ± 3.8	9.2 ± 2.5	73.6 ± 18.6	37.8 ± 10.9	12.3 ± 3.2
	12	68.1 ± 10.1	21.5 ± 4.2	10.4 ± 1.8	80.3 ± 21.7	49.2 ± 12.1	16.0 ± 3.5
Control group	0	60.6 ± 17.8	17.2 ± 3.6	9.1 ± 1.4	73.2 ± 17.2	37.5 ± 9.0	12.8 ± 2.8
	12	61.4 ± 13.7	18.6 ± 3.3	9.3 ± 2.8	72.6 ± 17.5	39.1 ± 12.7	13.2 ± 3.9

**TABLE 3:** Balance performance, exercise performance, and modified fall efficacy scale test results.

Group	Weeks	Total length (mm)	Elliptical area (mm^2^)	Total offset index (°)	TUG (s)	FTSST (s)	MFES (scores)
Elastic band group	0	942.3 ± 148.7	749.1 ± 108.6	3.65 ± 0.50	15.4 ± 2.21	12.9 ± 1.67	68.6 ± 9.3
	12	789.4 ± 134.5	642.9 ± 116.4	2.85 ± 0.44	12.8 ± 1.75	11.1 ± 1.50	80.7 ± 8.6
Vibration Group	0	940.0 ± 156.1	747.0 ± 100.6	3.70 ± 0.76	15.5 ± 1.93	13.0 ± 1.23	68.0 ± 11.5
	12	800.6 ± 156.2	655.7 ± 89.2	3.0 ± 0.63	13.6 ± 1.70	11.5 ± 1.40	79.0 ± 9.7
Elastic band plus vibration group	0	946.6 ± 187.3	752.4 ± 93.9	3.68 ± 0.54	15.2 ± 1.86	13.1 ± 1.53	67.9 ± 8.0
	12	676.0 ± 129.8	567.1 ± 85.3	2.27 ± 0.52	11.8 ± 1.51	9.3 ± 1.42	96.5 ± 13.7
Control group	0	938.9 ± 142.2	745.7 ± 91.8	3.72 ± 0.59	15.7 ± 1.85	12.7 ± 1.25	68.4 ± 8.3
	12	907.8 ± 180.0	726.4 ± 75.5	3.63 ± 0.48	15.6 ± 1.48	12.6 ± 0.78	66.8 ± 10.1

### Normality and interaction effects

Shapiro–Wilk tests confirmed normal data distribution. Two-way ANOVA revealed significant group × time interactions for balance parameters (TL: F = 5.184, *p* = 0.002; EA: F = 6.433, *p* < 0.001; TAI: F = 11.478, *p* < 0.001), functional performance (TUG: F = 7.248, *p* < 0.001; FTSST: F = 14.812, *p* < 0.001), and fear of falling (MFES: F = 19.734, *p* < 0.001). No interaction effects were observed for lower limb isokinetic muscle strength measures (*p* > 0.05).

### Between-group comparisons at 12 weeks

At week 12, hip flexion or extension and ankle flexion peak torque were not different among the groups (*p* > 0.05). Compared with the elastic band plus vibration group, the vibration group and control group showed a decrease in knee flexion peak torque (*p* = 0.049; *p* = 0.012), the elastic band group and control group showed a decrease in knee extension peak torque (*p* = 0.047; *p* = 0.003) and the control group showed a decrease in ankle extension peak torque (*p* = 0.034). The elastic band group showed decreases in TL (*p* = 0.005), EA (*p* = 0.005), TAI (*p* < 0.001), TUG (*p* = 0.026) and FTSST (*p* < 0.001) and an increase in MFES (*p* < 0.001). The vibration group showed decreases in TL (*p* = 0.011), EA (*p* = 0.001), TAI (*p* < 0.001), TUG (*p* < 0.001) and FTSST (*p* < 0.001) and an increase in MFES (*p* < 0.001). The control group showed decreases in TL, EA, TAI, TUG and FTSST (*p* < 0.001) and an increase in MFES (*p* < 0.001). Compared with the control group, the elastic band group showed decreases in TL (*p* = 0.005), EA (*p* = 0.002), TAI (*p* < 0.001), TUG (*p* < 0.001) and FTSST (*p* < 0.001) and an increase in MFES (*p* < 0.001); the vibration group showed decreases in TL (*p* = 0.011), EA (*p* = 0.007), TAI (*p* < 0.001), TUG (*p* < 0.001) and FTSST (*p* = 0.007) and an increase in MFES (*p* < 0.001).

### Within-group changes from baseline

Within-group comparisons were conducted between baseline and week 12. In the control group, no differences for all variables were observed (*p* > 0.05). The elastic band group showed 9.7% increase in knee flexion peak torque (*p* = 0.045, Cohen’s d = 1.25); reduction in TL (*p* < 0.001, Cohen’s d = 1.10), EA (*p* = 0.002, Cohen’s d = 0.96), TAI (*p* < 0.001, Cohen’s d = 1.73), TUG (*p* < 0.001, Cohen’s d = 1.34) and FTSST (*p* < 0.001, Cohen’s d = 1.08); and an increase in MFES by 17.6% (*p* < 0.001, Cohen’s d = 1.39); In the vibration group, knee extension peak torque increased by 16.3% (*p* = 0.011, Cohen’s d = 0.75). Additionally, reductions were observed in TL (*p* = 0.002, Cohen’s d = 0.95), EA (*p* = 0.001, Cohen’s d = 0.98), TAI (*p* < 0.001, Cohen’s d = 1.20), TUG (*p* < 0.001, Cohen’s d = 1.06) and FTSST (*p* < 0.001, Cohen’s d = 1.34). Moreover, MFES increased by16.2% (*p* < 0.001, Cohen’s d = 1.01). The CIG showed increases in hip and knee flexion (*p* = 0.001, Cohen’s d = 1.00; *p* = 0.005, Cohen’s d = 0.85) and knee and ankle extension peak torque (*p* = 0.001, Cohen’s d = 1.02; *p* = 0.001, Cohen’s d = 0.97) by 19.5%, 18.8%, 30.2% and 30.1% respectively. Furthermore, reductions were observed in TL (Cohen’s d = 1.72), EA (Cohen’s d = 2.11), TAI (Cohen’s d = 2.72), TUG (Cohen’s d = 2.03) and FTSST (Cohen’s d = 2.57), which decreased by 28.5%, 24.6%, 38.3%, 22.4% and 29.0%, respectively (*p* < 0.001). Additionally, MFES increased by 42.1% (*p* < 0.001, Cohen’s d = 2.63).

## Discussion

This study aimed to compare differences in lower limb isokinetic muscle strength, balance ability and physical performance between EBRT and WBVT in older women with a history of falls and investigate whether a combined intervention exerts an additional effect.

After 12 weeks, compared with the control group that did not use elastic bands, the elastic band group showed improved TL, EA, TAI, TUG, FTSST and MFES in older women. This result indicated that EBRT can improve balance ability and physical performance in older women, reduce the fear of falling and subsequently decrease the risk of falls. However, EBRT significantly improved only the knee flexor muscle strength. Previous research reported the effects of EBRT on older individuals. Kwak et al. (2016) demonstrated that 30 weeks of EBRT improved balance ability, gait function and physical performance in older adults to prevent falls. Oesen et al. (2015) found that 24 weeks of EBRT can improve TUG and FTSST in older adults, reporting an increase in knee extension muscle strength at 12 weeks but no significant changes in flexion strength; they found no significant changes in knee extension muscle strength from week 12 to week 24. A meta-analysis indicated that EBRT for 6–24 weeks improved muscle strength (grip strength, bench press performance, upper-limb traction force and knee extension muscle strength) in older adults (Martins et al., 2013). This study is aligned with previous research regarding improvement in balance ability and physical performance with EBRT (Kwak et al., 2016; Oesen et al., 2015). However, some discrepancies were observed regarding the effects on muscle strength (Oesen et al., 2015; Martins et al., 2013), which may be influenced by various factors, such as the intensity of elastic band resistance used, differences among participants and differences in methods or locations for assessing muscle strength. The improvement of balance ability and physical performance with EBRT may be attributed to the stimulation of proprioceptors in the joints to promote and consolidate proprioceptive conduction processes for rapidly sensing changes in body spatial position and maintaining balance (Kwak et al., 2016; Areas et al., 2013; Oesen et al., 2015). In this study, EBRT was performed in a squatting position with resistance from the lower extremities against the body mass and additional resistance from the elastic bands. The additional resistance from the elastic bands may contribute to the enhancement of balance and physical performance in older adults.

Similar to the control group without WBVT, the vibration group showed improvements in balance ability (static and dynamic) and physical performance in older women and a reduction in the fear of falling. The effects of WBVT on balance ability in older individuals remain controversial. For instance, 8 weeks of WBVT improved single leg standing time and centre of pressure velocity in patients who suffered chronic stroke (van Nes et al., 2004). A 6-week WBVT intervention in older individuals with an average age of 82 years showed improvements in static balance ability during seated position (Sitjà et al., 2015). However, some studies have presented different viewpoints. Hiroshige et al. (Hiroshige et al., 2014) found no significant effects of 8 weeks of WBVT on body centre of pressure movement distance in older adults. Another study indicated that 24 weeks of WBVT did not significantly reduce the risk of falling in older women with an average age of 82.4 years (Santin et al., 2017). By contrast, the current study found positive effects of 12 weeks of WBVT on balance ability and physical performance in older women. The effect of WBVT on physical performance and fall risk in older individuals has been reported. Eight weeks (Kawanabe et al., 2007) and 24 weeks (Osugi et al., 2014) of WBVT can improve walking capacity and reduce fall risk in older adults. It is worth noting that this study found that 12 weeks of WBVT significantly improved only the knee extensor isokinetic muscle strength of older women. This discrepancy with previous research might be due to differences in participant selection, measurement methods for assessing muscle strength and intervention duration. The mechanism by which WBVT improves balance ability and physical performance may involve the activation of α-motor neurons at vibrated areas, strengthening muscle fibre recruitment capacity, promoting neurotransmitter secretion to stimulate tendons and improving muscle strength (van Nes et al., 2004). WBVT can activate proprioceptors, such as the Golgi tendon organ, promote the transmission of proprioceptive information in neural pathways and induce changes in the adaptability and neuromuscular excitability of the body, thereby enhancing performance (Van Heuvelen et al., 2021; Sá et al., 2023).

This study demonstrated that combined intervention further improves balance ability and physical performance and reduces the fear of falling in older women with a history of falls, thereby reducing the risk of recurrent falls. WBVT combined with KAATSU training was found to have a greater effect on improving lower limb isokinetic muscle strength in older adults than single-mode training (Xiong and Liu, 2023). This study expands upon the research (Xiong and Liu, 2023), although the mechanisms underlying the additive effects of combined intervention are not yet clearly understood, they may be related to the synergistic effects. The effects of single-mode training (EBRT or WBVT) may be limited because of physiological decline in systems, such as reduced muscle reaction speed in older individuals (Gonçalves et al., 2023). However, when older adults engage in EBRT and WBVT simultaneously, α-motor neurons and proprioceptors are quickly activated, and their physical performance is enhanced. Additionally, increased physical performance can reduce the fear of falling in older individuals (Schoene et al., 2019; Chang et al., 2024b). Strengths of this study include the randomized controlled design, the use of validated functional tests (e.g., TUG, FTSST), and the inclusion of older women with a history of falls, a population at high risk for recurrent falls. Additionally, the combination of EBRT and WBVT provides novel insights into synergistic effects on isokinetic muscle strength and balance.

The present study acknowledges several inherent limitations that warrant further exploration and address in future research endeavors. Initially, the participant pool was confined to an older female demographic from a single community, which introduces a potential bias and restricts the generalizability of our findings to other populations. To mitigate this limitation, subsequent studies should aim to recruit a more diverse and representative sample that spans various age groups, genders, and socioeconomic backgrounds, thereby enhancing the external validity of the research outcomes. Moreover, the study’s intervention duration of 12 weeks, while sufficient for preliminary insights, may not be adequate to capture the full spectrum of long-term effects and the sustainability of the interventions. Additionally, the elastic band resistance intensity was standardized based on absolute band length (2× original length) but not individualized relative to participants’ strength capacity, which may limit the generalizability of the results. To address this, future research should extend the intervention period and incorporate long-term follow-up assessments. This approach will provide a more comprehensive understanding of the sustained impact of combined interventions on postural control and fall prevention in older adults. Additionally, the current study did not account for a range of variables that could significantly influence postural control capabilities, such as lower limb proprioception and neuromuscular response times. Future investigations should incorporate a multifactorial assessment, including these and other relevant factors, to offer a more holistic view of the efficacy of combined interventions in older populations with a history of falls.

## Conclusion

After 12 weeks of intervention, the combination of EBRT and WBVT can enhance knee and ankle isokinetic muscle strength and further improve balance ability and physical performance and reduce fear of falls in comparison with single interventions. EBRT and WBVT had limited effects on improving lower limb muscle strength but enhanced balance ability, physical performance, and reduced fear of falls in older women with a history of falls. Both training modalities showed similar effects.

## Data Availability

The raw data supporting the conclusion of this article will be made available by the authors, without undue reservation.
